# Gas chemical adsorption characterization of lanthanide hexafluoroacetylacetonates

**DOI:** 10.1007/s10967-017-5232-z

**Published:** 2017-03-21

**Authors:** S. Adam Stratz, Steven J. Jones, Austin D. Mullen, Manny Mathuthu, Colton J. Oldham, John D. Auxier, Howard L. Hall

**Affiliations:** 1 Department of Nuclear Engineering, University of Tennessee, 315 Pasqua Engineering Bldg., Knoxville, TN 37996 USA; 2 Radiochemistry Center of Excellence, University of Tennessee, 1508 Middle Dr., Knoxville, TN 37996 USA; 3 Bredeson Center for Interdisciplinary Research, University of Tennessee, 1640 Cumberland Ave., Knoxville, TN 37996 USA; 4 Center for Applied Radiation Science and Technology (CARST), North-West University (Mafikeng), Cnr Albert Luthuli Road & University Drive, Mmabatho, Mafikeng, 2735 South Africa; 5 Institute for Nuclear Security, University of Tennessee, 1640 Cumberland Ave., Knoxville, TN 37996 USA

**Keywords:** Nuclear forensics, Hexafluoroacetylacetone, Rare earth separations, Nuclear security, Post-detonation

## Abstract

Newly-established adsorption enthalpy and entropy values of 12 lanthanide hexafluoroacetylacetonates, denoted Ln[hfac]_4_, along with the experimental and theoretical methodology used to obtain these values, are presented for the first time. The results of this work can be used in conjunction with theoretical modeling techniques to optimize a large-scale gas-phase separation experiment using isothermal chromatography. The results to date indicate average adsorption enthalpy and entropy values of the 12 Ln[hfac]_4_ complexes ranging from −33 to −139 kJ/mol K and −299 to −557 J/mol, respectively.

## Introduction

Rising nuclear security concerns surrounding the safety and proliferation of nuclear materials used in nuclear power plants, in addition to possible diversion of materials for illicit purposes by nuclear terrorists, calls for the development of novel technologies for rapid response to such potential threats. The heavy fission product spectrum is largely comprised of lanthanide elements which have been subsequently used by many researchers as characteristic nuclear forensic signatures of fissile (e.g., uranium, plutonium) materials that could be targeted by terrorists. An investigation of lanthanide chelate adsorption characteristics provides important information contributing to pre- and post-detonation nuclear forensic techniques. Previous work has detailed the importance of these values as they pertain to post-detonation nuclear forensics [1]. We formerly established the theoretical plausibility of gas-phase separations using lanthanide chlorides in Monte Carlo simulations [2, 3], and afterward demonstrated successful detection of Ln[hfac]_4_ complexes using a gas chromatography (GC) instrument coupled to an inductively-coupled plasma time-of-flight mass spectrometer (ICP-TOF-MS) [4]. We now present experimentally-determined adsorption enthalpies that can be used in a theoretical model to optimize an isothermal gas-phase separation experiment using the GC–ICP-TOF-MS setup. Following established adsorption enthalpy methodology using temperature ramping in a chromatographic system, Ln[hfac]_4_ deposition patterning in a quartz column enabled the convergence of these valuable thermodynamic values.

## Experimental method

If a suite of complexes, in this case the Ln[hfac]_4_ complexes, demonstrate variation in volatilization temperatures, a temperature gradient or ramped temperature chromatography method can be used to experimentally determine adsorptive thermodynamic characteristics. Each complex will elute from the column at a characteristic temperature directly correlated to its thermodynamic properties. This temperature can be used in conjunction with experimental operating conditions to converge the enthalpy and entropy of adsorption of the complexes.

Steffen and Bachmann [5] have outlined a derivation used to calculate the entropy and enthalpy of adsorption from deposition patterns within a temperature gradient. The equation resulting from this derivation is described in (1) as:

1 $$ \log t_{\text{a}} = \frac{{ - \Updelta H^{ \circ } }}{{2.3RT_{\text{a}} }} + \frac{{\Updelta S^{ \circ } }}{2.3R} + \log \frac{{RsT_{0} }}{{au_{0} V_{\text{g}} }}, $$

where *t*_a_ is the time of adsorption, Δ*H*° is the enthalpy of adsorption (unknown), *R* is the ideal gas constant, *T*_a_ is the adsorption temperature, Δ*S*° is the entropy of adsorption (unknown), *s* is the open surface area of the column per unit length, *T*_0_ is the original temperature, *a* is the temperature gradient, *u*_0_ is the linear flow velocity, and *V*_0_ is the open volume of the column per unit length.

When several experiments are performed at various operating conditions (changing, for example, the linear gas velocity *u*_0_), and the resulting lines derived from the above equation are plotted on a graph of enthalpy of adsorption versus entropy of adsorption, the intersection of the lines yields the resulting enthalpy and entropy of adsorption of the complex under interrogation.

In lieu of a temperature gradient, a procedure using cold column complex deposition and temperature ramping was used to mimic the conditions a temperature gradient provides. One end of the quartz column was introduced to room temperature conditions while the other end remained connected to the injection port of the GC instrument, where the injected sample could be flash vaporized. The majority of the column was within the GC oven where the temperature could be easily controlled with only the last 5–6 cm of column remaining in room temperature conditions. A given Ln[hfac]_4_ complex was injected at a sufficiently high temperature such that it traversed the length of the column and deposited on the last 5–6 cm where it was exposed to a sudden negative temperature gradient. The end with the complex deposit was then coiled into a cooled GC with the remainder of the column and subjected to a gradual temperature increase. Each temperature point was held for 10 min, after which the column was inspected for remaining deposition. The temperature was increased until the deposit eluted within the 10-min timeframe. Using this method, we were able to confine a 5-degree window (or less) in which the complex would elute; this temperature is the temperature at which the complex becomes a gas and elutes at the given operational conditions, and conversely, the deposition temperature at which the complex condenses from a gas to a solid within the column. A temperature gradient provides the same information this method produces; namely, both methods allow the deposition temperature to be measured using experimental parameters. Using this deposition range and known operating conditions, *T*_0_ and *T*_a_ were assumed to be the beginning and end of the measured deposition temperature range, while *t*_a_ was assigned a value that allows the complex to adsorb at the midpoint of this range, giving a deposition temperature equal to the midpoint of the experimental temperature deposition values.

With the known conditions described above in conjunction with other operating conditions, all necessary variables can be substituted into the derived equation and plotted to yield linear equations. Performing the same experiment with the same complex, but varying the carrier gas flow rate, produces a line with a slightly different slope that intersects the first line under the original operating conditions. This intersection yields the enthalpy and entropy of adsorption values. The more experiments performed at varied conditions for a given complex, and subsequently the more lines plotted for those experiments, the more the error can be reduced and mitigated.

## Results

Samples of individual Ln[hfac]_4_ complexes, aside from La[hfac]_4_, Ce[hfac]_4_, and Pm[hfac]_4_, were injected according to the methodology outlined in the previous chapter to observe and isolate deposition temperature ranges along the column. Four pressure values were initially used to discern temperature variances as they related to column pressure; however, after injecting several samples at the highest pressure value of 42 psi, it was observed that the adsorption properties of the complexes were completely overridden by the high pressure within the column. A minimum tank pressure of 10 psi (physical limitation) and maximum pressure of 42 psi (thermodynamic limitation) left an acceptable range of three individual pressures: 12, 22, and 33 psi, to be used. Adding more pressure values within this range did not allow for sufficient discernment of deposition temperature ranges between the pressure values, so in the end, only these three pressure values were used to measure the adsorption properties of the complexes. Resulting deposition temperature ranges are shown in Table 1.

**Table 1:** Pressure-dependent raw deposition temperatures of Ln[hfac]_4_ complexes (°C)

	12 Psi	22 Psi	33 Psi	42 Psi
Pr[hfac]_4_	140–145	135–140	130–135	115–120
Nd[hfac]_4_	140–145	135–138	130–135	111–117
Sm[hfac]_4_	150–155	142–148	130–135	104–109
Eu[hfac]_4_	155–160	143–148	113–118	113–116
Gd[hfac]_4_	150–155	125–130	110–112	105–110
Tb[hfac]_4_	110–115	96–100	92–96	–
Dy[hfac]_4_	126–130	120–125	115–120	–
Ho[hfac]_4_	122–127	117–122	110–115	–
Er[hfac]_4_	145–150	120–125	107–112	–
Tm[hfac]_4_	140–145	131–136	110–115	–
Yb[hfac]_4_	155–160	129–134	110–115	–
Lu[hfac]_4_	145–150	130–1350	105–110	–

**Fig. 1:**
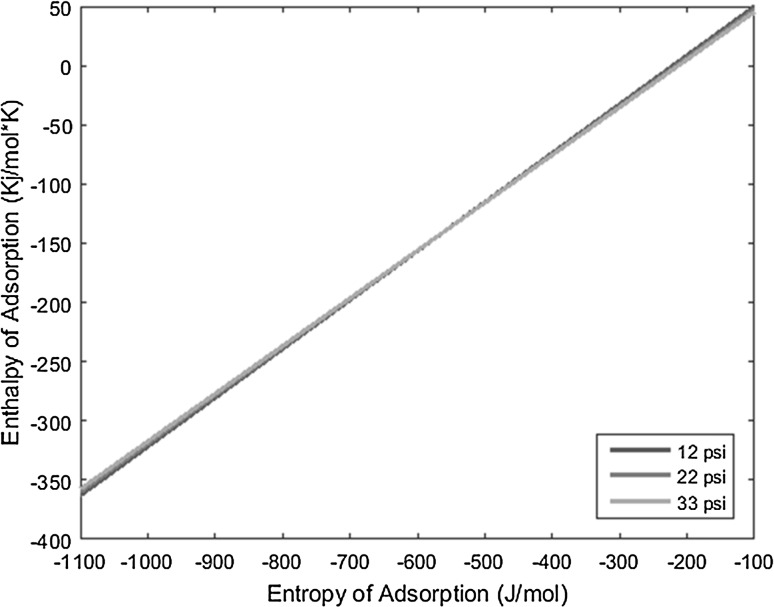
Pr[hfac]_4_ adsorption convergence plot

**Fig. 2:**
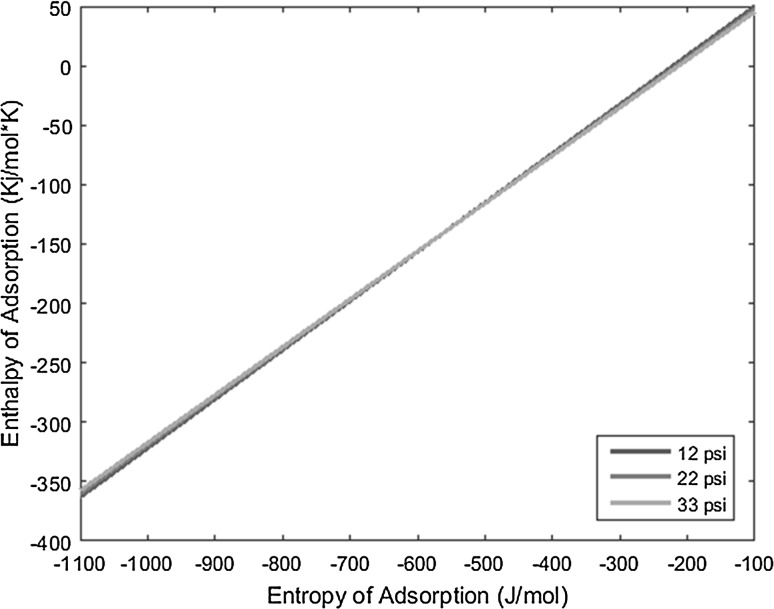
Nd[hfac]_4_ adsorption convergence plot

**Fig. 3:**
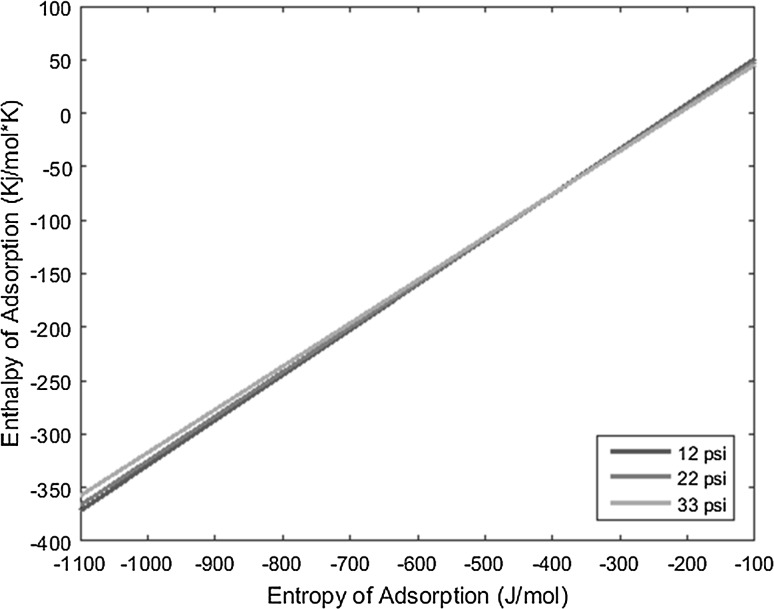
Sm[hfac]_4_ adsorption convergence plot

**Fig. 4:**
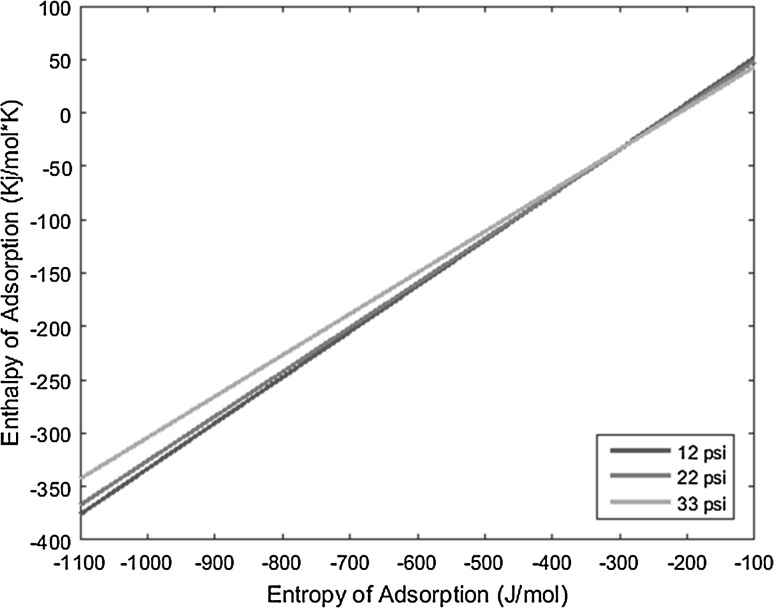
Eu[hfac]_4_ adsorption convergence plot

**Fig. 5:**
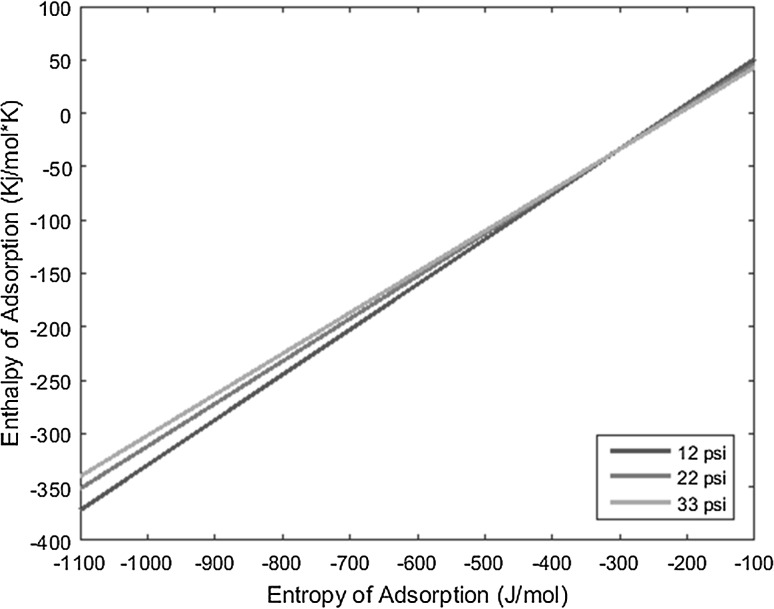
Gd[hfac]_4_ adsorption convergence plot

**Fig. 6:**
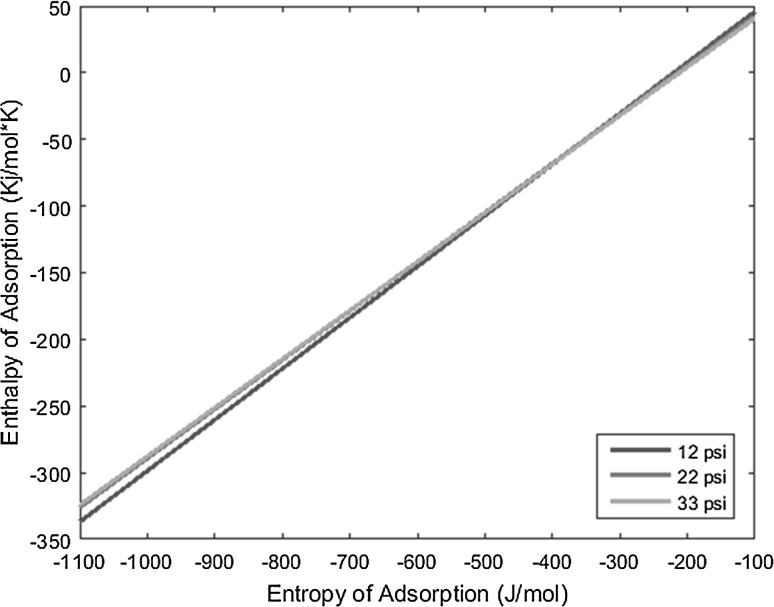
Tb[hfac]_4_ adsorption convergence plot

**Fig. 7:**
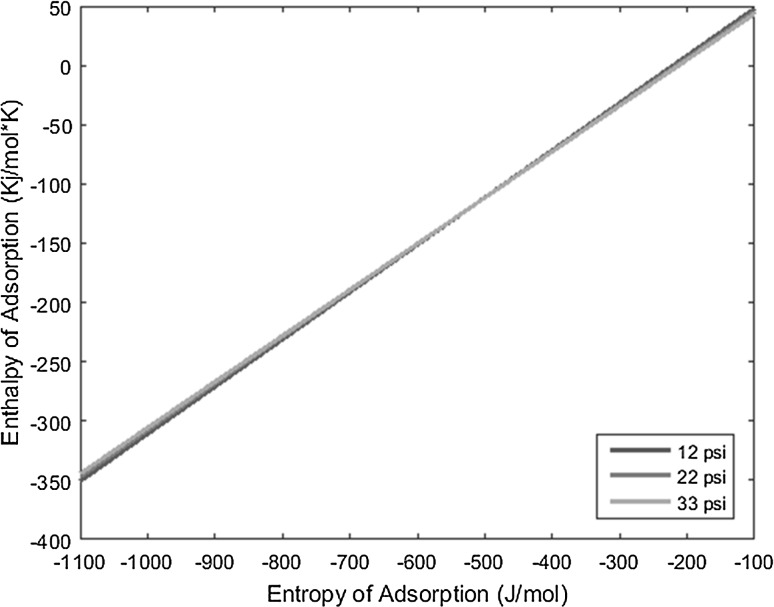
Dy[hfac]_4_ adsorption convergence plot

**Fig. 8:**
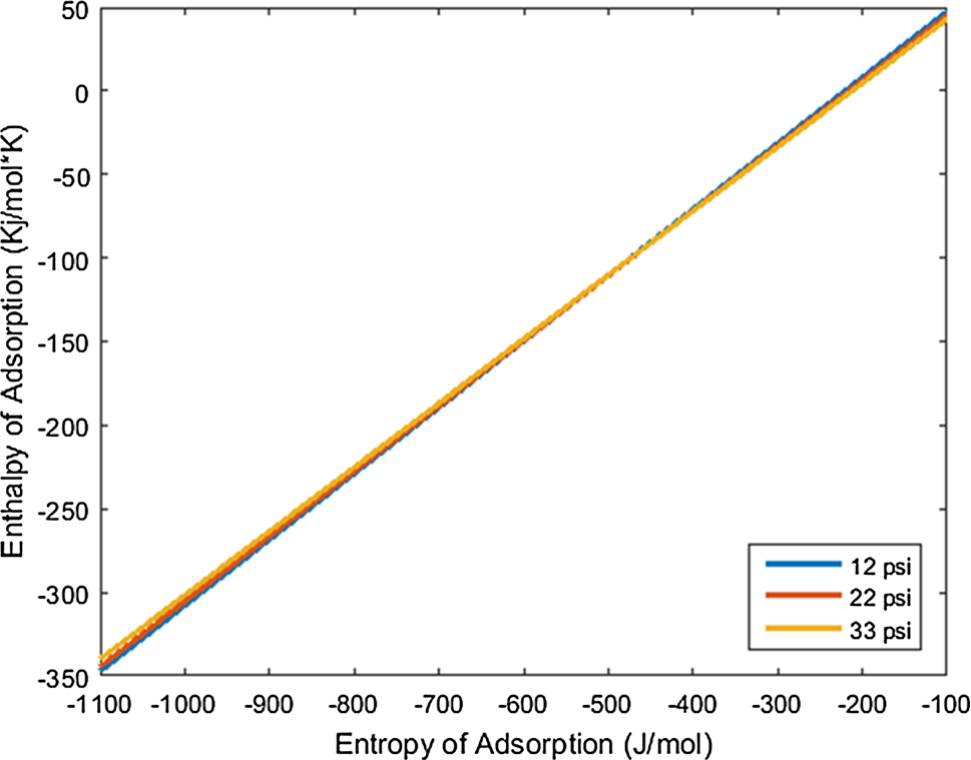
Ho[hfac]_4_ adsorption convergence plot

**Fig. 9:**
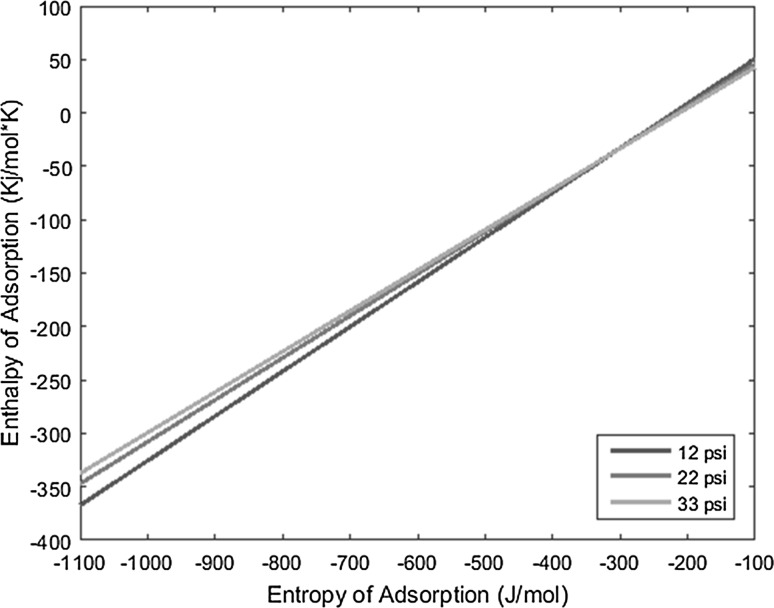
Er[hfac]_4_ adsorption convergence plot

**Fig. 10:**
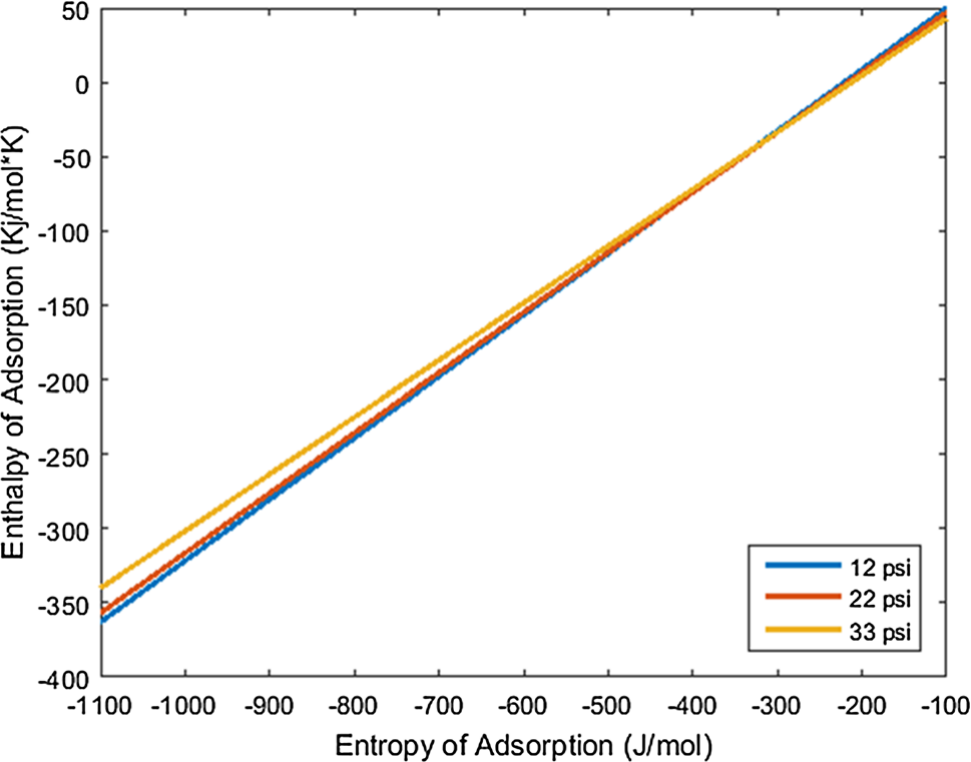
Tm[hfac]_4_ adsorption convergence plot

**Fig. 11:**
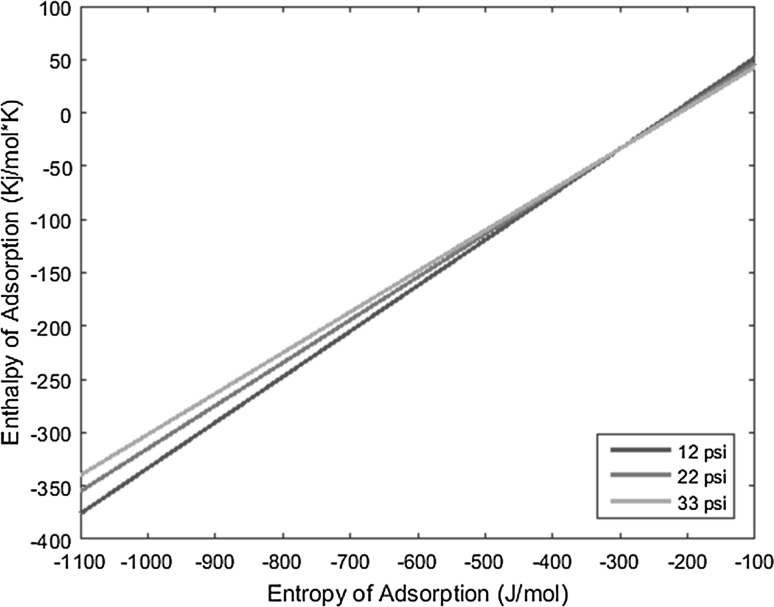
Yb[hfac]_4_ adsorption convergence plot

**Fig. 12:**
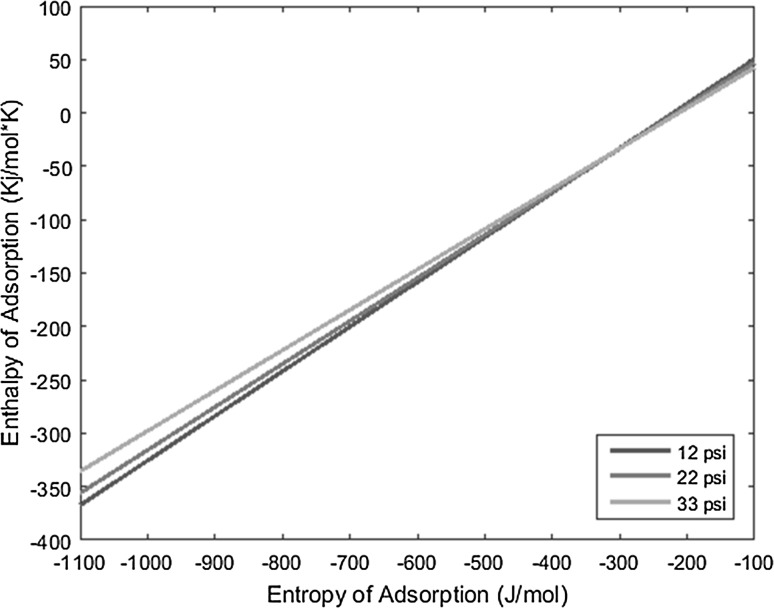
Lu[hfac]_4_ adsorption convergence plot

The temperature deposition profiles of each element were used to model adsorption behavior using the equation outlined in the previous section. In conjunction with pressure, deposition temperature, flow rate, and other operating conditions, adsorption enthalpy and entropy values could be plotted at each temperature value and graphed concurrently. The three points of intersection of these lines (produced from the three pressure values used during experimentation and subsequent variations in deposition temperature) relay three converged entropy and enthalpy values for each complex. Under ideal conditions, these points would all overlap and convey a single value for enthalpy and entropy of adsorption. However, due to experimental error, the three points of intersection must be averaged. Figures 1, 2, 3, 4, 5, 6, 7, 8, 9, 10, 11 and 12 show the plots resulting from the data obtained in Table 1.

The points of intersection of the lines shown in Figs. 1, 2, 3, 4, 5, 6, 7, 8, 9, 10, 11 and 12 are averaged and indicated in Table 2, along with the standard deviation from both experimental and theoretical error. Figures 13 and 14 show these values graphically.

**Table 2:** Enthalpy and entropy of adsorption values of Ln[hfac]_4_ complexes

	−∆*H*_ads_ (−kJ/mol K)	−∆*S*_ads_ (−J/mol)
Pr	139 ± 4	557 ± 19
Nd	139 ± 5	557 ± 22
Sm	76 ± 17	398 ± 51
Eu	42 ± 21	317 ± 59
Gd	38 ± 7	310 ± 27
Tb	83 ± 49	440 ± 143
Dy	118 ± 8	516 ± 29
Ho	109 ± 21	493 ± 66
Er	39 ± 10	315 ± 34
Tm	53 ± 25	348 ± 70
Yb	33 ± 3	299 ± 17
Lu	38 ± 11	310 ± 38

**Fig. 13:**
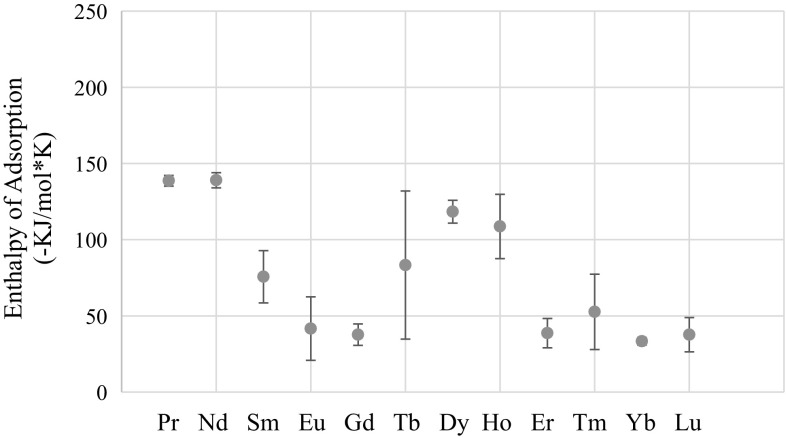
Enthalpy of adsorption of Ln[hfac]_4_ complexes

**Fig. 14:**
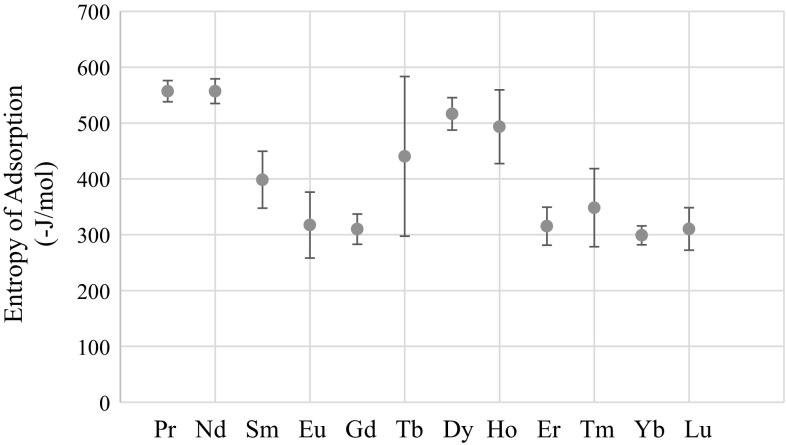
Entropy of adsorption of Ln[hfac]_4_ complexes

## Conclusions

The enthalpy and entropy of adsorption of 12 lanthanide hexafluoroacetylacetone chelates have been experimentally measured for the first time. These values were determined using a modified temperature gradient method to observe deposition temperature patterning along an uncoated quartz column in a variety of operational conditions. These experimentally-determined thermodynamic values are vital input parameters for theoretical models to optimize experimental conditions for a large-scale gas-phase separation of heavy fission product chelates using hexafluoroacetylacetone. Even if the measured thermodynamic values, once used in the model, do not result in predicted separations in the gas phase using this ligand, these adsorption values remain valuable to the thermodynamic community to corroborate other thermodynamic models using volatile lanthanide complexes.
